# The Role of Transformational Leadership on Firm Performance: Mediating Effect of Corporate Sustainability and Moderating Effect of Knowledge-Sharing

**DOI:** 10.3389/fpsyg.2022.883224

**Published:** 2022-07-07

**Authors:** Muhammad Asim Shahzad, Tahir Iqbal, Naveed Jan, Muhammad Zahid

**Affiliations:** ^1^Department of Business Administration, North China Electric Power University, Beijing, China; ^2^Department of Business Administration, University of Zaragoza, Zaragoza, Spain; ^3^Department of Management Science and Engineering, Shandong Normal University, Jinan, China; ^4^Department of Management Sciences, City University of Science and Information Technology, Peshawar, Pakistan

**Keywords:** firm performance, transformational leadership, corporate sustainability, knowledge-sharing, management

## Abstract

The primary purpose of the research is to investigate the mediating role of corporate sustainability in the relationship between the impacts of transformational leadership on the performance of firms. This study also aimed to investigate the moderating role of knowledge-sharing on the relationship of transformational leadership with corporate sustainability. Respondents of the study were the top management of large Chinese automobile sectors, such as Shanghai Automotive Business Corporation (Group), China FAW Group Corporation, Dongfeng Motor Co., Ltd., Beijing Automotive Group Co., Ltd., and China North Industries Group Corporation. These are the companies with the biggest market share in the automobile manufacturing industry in China. The data was gathered by using a self-administrative survey questionnaire from 198 individuals operating in different automobile industries in different sectors of China. The data were analyzed using structural equation modeling (SEM) through the Smart PLS 3.3.2 software. The results of this study revealed that transformational leadership has a positive and significant effect on the performance of the firm. Corporate sustainability has a significant positive mediating role in the association of transformational leadership and firm performance. Findings indicated that knowledge-sharing also has a positive moderating role in the association between transformational leadership and firm performance. The findings of this study contribute to the body of knowledge and show that leadership style has a significant effect on firm performance and that knowledge-sharing culture in firms is essential for better performance of the firm. Furthermore, firms may improve their performance by improving their sustainability and by creating knowledge-sharing culture. The findings are important, particularly in connection with a developed country like China. The findings have important insights for various stakeholders, i.e., government, regulatory bodies, practitioners, academia, industry, and researchers.

## Introduction

In the contemporary rapidly changing business environment, it is highly important to understand the factors that influence the performance of businesses (Sadeghi and Pihie, 2012). In response to intense rivalry, advancements in technologies, and fast-changing demands of the customer, firms are required to implement practices effectively to meet targets and exceed performance (Mammassis and Kostopoulos, 2019). Still, whether the performance of the organization is influenced, to some extent, by leadership style and behavior is a debatable question. As a reference, Starbucks was made the most popular brand in the world by Howard Schultz, and Burberry’s revenue was doubled in 5 years by Ahrendts (2013). These examples suggest that management style positively influences the performance of a company. In contrast, some researchers concluded negative implications from having popular CEOs, and that appointing a megastar chief executive officer with a charismatic personality has no influence on the effectiveness of organizations, but only enhances pay expenditure (Chen et al., 2021) and promotes a blind following with potentially negative consequences, seen in the case of Jeff Skilling’s role in the demise of Enron (Idris et al., 2022). In the literature, researchers highlighted numerous variables that potentially influence the performance of the business. These variables include entrepreneurial orientation (Patel et al., 2015), information technology (Chae et al., 2018), and business strategy (Tarí et al., 2017). Despite many variables influencing firm performance, leadership style has a significant contribution to performance. In addition, leaders importantly influence the policies of a business that ultimately shape the competitive environment (Bass and Avolio, 1994). Multiple challenges are faced by an organization in an environment of intense competitiveness. Therefore, the primary challenge for a business is to obtain a competitive advantage by developing suitable strategies for better operational performance (Mulki et al., 2015).

Previously, financial performance was the key element of focus for organizations. In the modern business environment, information development and its competitive basis are transformed into intangible resources, and tangible financial outcomes are transformed into leadership performance (Burhan and Rahmanti, 2012). Consequently, non-financial indicators should also include factors such as quality and customer satisfaction, which may be of use when evaluating the operational performance of a business by incorporating competitive position (Orji, 2019). If an organization intended to improve its performance, the leadership style of the executive will have a significant contribution to the overall operational performance of an entity (Nagendra and Farooqui, 2016). Most of the literature on the topics of leadership and business performance focused on the impact of leadership style on organizational performance and development (Victoria et al., 2021). However, few researchers studied the association between leadership style, the performance of the business, and strategies for managing human resources (Zula and Chermack, 2007). While implementing managerial activities, leadership style may be a crucial element for smooth progress. After the emergence of firm resource-based views, human resources are now considered the most significant component in gaining a competitive edge and achieving organizational goals (Barney and Mackey, 2016).

In addition, under current contextual pressures, organizations are facing numerous management-associated challenges, including extreme rivalry among competitors, shortage of resources, more rational, demanding customers, rapid advancements in technologies, changing climate, and pressure from stakeholders (Thangaveloo et al., 2022). In long-term settings, a systems perspective is needed to attain financial, ecological, and social outcomes (Nirino et al., 2019). Many paths are suggested by researchers to attain these goals, including the development of particular models and frameworks for managing sustainability (Balugani et al., 2020). Integration of sustainability is required at strategic and operational levels of management by keeping in view the future and current targets of stakeholders (Awan et al., 2017). With the development of leadership models in modern scenarios, visionary leaders emerged and became popular among leadership researchers (Harsanto and Roelfsema, 2015). Leadership paradigms are shifting from traditional to visionary, also termed “charismatic,” “transformational,” or “inspirational” leadership, which also considers the emotional dimensions of the phenomenon (Laeeque and Babar, 2016). Asif et al. (2019) argued that transformational leaders uplift the contribution of team members to accept the mission and purpose of the organization by motivating them to sacrifice their interests and achieve the common interest of the group. Moreover, visionary leadership transforms individual desires, doctrines, preferences, and ambitions into shared and common interests by sharing values, vision, collective decision-making, and authorization (Zhou et al., 2018).

Furthermore, followers of transformational leadership are empowered and work independently for a collective purpose. They are inspired by the charisma and vision of their leaders (Jing et al., 2020). Additionally, corporate sustainability is enhanced by transformational leaders that eventually influence the performance of an organization (Gerard et al., 2017). A transformational leadership behavior in a knowledge-sharing environment integrates and supports models of strategy implementation successfully. Transformational leadership behavior is suitable for the implementation of strategy because it creates an environment where participants have confidence and respect for their leader or manager, which motivates them to do more than expected (Zhang et al., 2015; Zhang S. et al., 2019; Zhang W. et al., 2019). Moreover, knowledge-sharing directs businesses to avail newly emerged opportunities in the market (Wang et al., 2016).

In the comprehensive study of the particular frameworks that integrate knowledge-sharing and transformational leadership, this research examines the role of transformational leadership on the performance of business by moderating the role of knowledge-sharing. This study will contribute to the stream of literature in many ways. Firstly, from a theoretical perspective, this is a pioneering study on the transformational style of leadership of top executives about the organizational performance with the moderating role of knowledge-sharing. This study also contributes to the literature by addressing the question of how knowledge-sharing jointly forms an integration that helps in the effective transformation of several behaviors of transformational leadership into the higher performance of the business. This discussion leads to the following three research questions:

RQ1: How does effective transformational leadership lead to higher firm performance?
RQ2: What is the mediating role of corporate sustainability in the relationship between the impacts of transformational leadership on the performance of firms?
RQ3: What is the moderating role of knowledge-sharing in the relationship between the impacts of transformational leadership on the performance of firms?

A brief review of literature on the role of transformational leadership on firm performance mediating the effect of corporate sustainability and the moderating effect of knowledge-sharing is presented in the section after that, which is then it followed by the development hypothesis of the study. Then, the method used in the study is effectively designated. After the description of analysis and results, a comprehensive discussion of the research findings containing the study’s practical implications and practices was consequently performed. The last section of the research highlights the confines of the study, along with recommendations for further study for various researchers and scholars.

## Literature Review

### Theoretical Framework and Hypothesis Development

#### Leader-Member Exchange Theory

The phrase “vertical dyad linkage” (VDL) was first used to describe the leader-member exchange concept (De Clerck et al., 2021). According to Hanasono (2017) the leader-member exchange concept is separated into four stages, each of which is connected to and builds on the preceding stages (Hanasono, 2017). According to LMX theorists, organizational leaders should give their followers more power, foster the sharing of work-related knowledge, and allow participation in decision-making processes (Jiang and Yang, 2015). The leader-member exchange theory by Herman and Mitchell (2010) is a psychological process variable that serves as a bridge between transformational leadership and knowledge management. Transformational leadership attributes are predictive of the leader-member exchange (LMX) paradigm (Hanasono, 2017). The leader-member exchange concept identifies organizational personnel’s unique responsibilities, interpersonal interactions, and related functions (Graen and Uhl-Bien, 1995). According to transformational leadership theory, leaders assure organizational success by passing on the organization’s vision, mission, and goals to their followers (Burch and Guarana, 2014). As a result, the leader-member exchange theory implies that leaders and their followers build mutual norms and social exchanges (Jiang and Yang, 2015).

### Social Learning Theory

The social learning theory explains that individuals learn in organizations through monitoring their own and others’ behavior (McLeod, 2016). By studying their leaders and their intellectual stimulation, followers learn how to think creatively and come up with methods for creating new ideas (Mittal and Dhar, 2015). Employees can notice the individualized consideration of transformational leaders by observing the information-sharing given in the organization, which favors new ideas (Clarke, 2013). This knowledge-sharing procedure can assist employees in developing creativity and motivating them to set challenging goals (Wade and Hulland, 2004). Employees’ motivation for creativity can be increased by transformational leaders and this process leads to the development of creative self-efficacy (Koh et al., 2019). This can help to reduce obstacles at work, motivate employees for creative pursuits, and develop higher creative performance (Koh et al., 2019).

### Transformational Leadership Theory

The transformative leadership theory was first articulated by Idris et al. (2022). Leadership focuses on addressing fundamental requirements and higher needs, while inspiring followers to come up with new-fangled thoughts and make the workplace a better place to work (Ghasabeh et al., 2015). According to Clarke (2013) and Clarke and Braun (2013) transformational leadership has four dimensions. These are idealized influence, tailored consideration, intellectual stimulation, and motivational inspiration (Fellows et al., 2003). In the last 30 years, many adjustments were made to the transformational theory of leadership. Nowadays, these kinds of leaders are defined by researchers as leaders who influence and encourage followers to sacrifice their interests at the price of collective interests (Banks et al., 2016). Despite criticism of the application of transformational leadership, Van Knippenberg and Sitkin (2013) categorize the influence of this leadership style into four sub-dimensions (Deinert et al., 2015).

#### Idealized Influence

The idealized influence leadership style focuses on the basic purpose of idealized influence to create a common vision and strengthen relationships with followers (Sayyadi and Provitera, 2016). Idealized influence states that leaders act as role models for followers and receive acknowledgment, admiration, and trust from them (Phaneuf et al., 2016).

#### Individualized Consideration

The individualized consideration leadership style focuses on understanding the specific requirements of employees and inspiring followers to create a learning environment at the corporate level to mobilize their support for organizational goals (Ghasabeh et al., 2015). Individualized consideration is the effort of a leader in admitting the needs of individuals and providing guidance to them as a counselor or trainer. Some of the researchers recommended the overall application of transformational leadership in operations (Anderson and Sun, 2015).

#### Intellectual Simulation

Intellectual stimulation is the degree to which a leader can create an innovative environment for solving problems (Jin et al., 2016). The Intellectual stimulation leadership style encourages employees to share their knowledge to develop more inventive thoughts and justifications.

#### Inspirational Motivation

Inspirational motivation is the competence of a leader in communicating a convincing mission with compelling future perspectives that ultimately elevate the efforts and spirit of the employees with the courage to face challenges. The Inspirational motivation leadership style focuses on motivating human assets to achieve better levels of the desired potential. These four dimensions characterize an effective leader in a knowledge-driven economy built on producing and managing intellectual capital within firms or businesses.

### Chinese Context

Transformational leadership encompasses a diverse set of characteristics that are influenced by several cultural elements, as well as basic workplace environments. Many academics have developed comparable scales depending on their cultural backgrounds. Chinese researchers have also looked into transformational leadership in the context of Chinese culture and corporate organizations. Li et al. (2015) created a new Chinese transformational leadership paradigm based on their analysis of Chinese business sectors. Transformational leadership is typically thought of as a style of leadership that can help both leaders and followers enhance their morality and maturity. Chinese researchers have confirmed and extended the transformational leadership concepts as proposed by Liu et al. (2010). Based on Bass’ concepts, as well as their analysis of Chinese business sectors, Li et al. (2015) established a new Chinese transformational leadership model. To explore the structure of transformational leadership theory and its link with leadership effectiveness, data were collected from a variety of sources and were evaluated using factor analysis, reliability analysis, and regression analysis. The study suggests that the use of a transformational leadership questionnaire showed good validity and reliability and it was appropriate for the Chinese culture. Kanwal et al. (2019) developed an integrated model to examine the validity of transformational leadership in a Chinese cultural environment, as well as the efficacy of authoritarian leadership in Chinese private firms. The findings of this study support the importance of transformational leadership in Chinese culture. Sun and Leithwood (2015) conducted a content study that showed transformational leadership in China is divided into eight distinct categories. According to exploratory factor analysis (EFA) in China, transformational leadership is built around four dimensions that encompass ethical show off, captivation, visioning, and personalization.

### Hypotheses Development

#### Transformational Leadership and Performance

An individual’s or an organization’s performance is defined as the extent of work they put in and remain successful while achieving a mission. The concept is presented by the prominent employees of an organization when accomplishing their assigned tasks (Bass and Avolio, 1994). Accordingly, the success of an organization depends upon the capabilities of organization personnel (Yıldız et al., 2014). The performance of a business can be assessed by looking at fulfilled tasks and set targets or the aim of business at the end (Benligiray and Ahmet, 2017). The subjective, as well as objective, approaches can be applied to measure the performance of the business. In the literature, both the combined approaches are also applied to counter the drawbacks of each of the approaches. Evidence shows that profitability, market share, and sales are the most widely used indicators while measuring subjectively. However, while objective evaluations, ROA and ROE are common indicators for measuring firm performance (Yıldız and Karakaş, 2012). Literature indicates that numerous methods of performance evaluation are established by the researchers, but none of the single methods is valid in all contexts. Over the years, the transformational theory of leadership gained considerable support from researchers (Wang et al., 2011; Van Knippenberg and Sitkin, 2013). In the literature, the positive influence of transformational leadership is proven on various variables, including job satisfaction (Braun et al., 2013), commitment to the organization (Wang et al., 2011), innovation and creativity (Anderson et al., 2014), and well-being of employees.

By keeping in mind the supposition that transformational style impacts performance greatly, a large number of researchers evaluated the perspective of likely impacts of transformational leaders on performance (Khalili, 2017). A large number of research studies have been published on the meta-analyses of transformational theory and the performance of the business (Wang and Zhu, 2011). Furthermore, these analyses recommended a positive association between transformational leadership and various performance indicators that include followers’ insights of leader effectiveness, the job performance of the leader, sales volume, and profit ratios. While measuring performance at the organizational level, evaluation based on the financial data shows weaker relation of transformational leadership with performance indicators as compared to the evaluation based on the subjective measures among these variables (Sadeghi and Pihie, 2012). The transformational leadership model and trickle-down leadership frameworks concluded with numerous descriptions of the answers about why and how CEOs can impact the performance of their organization (Bass, 1999; Bass and Steidlmeier, 1999). Potentially, an influential leader can contribute to the success of the business by influencing direct reports of business in teams of top executives (Wang et al., 2017). It is anticipated that leaders, having a transformational style of leadership, play the character of an influencer for the effectiveness of organizational management at the level of subordinates, and as a result, their actions are elicited by transformational leaders that ultimately influence the performance of the firm (Wang et al., 2014).

About idealized influence or charisma, CEOs potentially form the performance of their organization by communicating its core values and beliefs, thus establishing a collective mission (Kim and Shin, 2019). While performing as role models, they provide appropriate structure and communicate to others their organizational expectations from them that consequently produce better overall performance (Jensen et al., 2020). Moreover, leaders are expected to enhance performance through inspirational motivation that provides meaning to the followers (Obeidat and Tarhini, 2016). Through this, followers are able to deal with the challenging environment and show a strong commitment to the achievement of organizational targets (Kim and Shin, 2019). In this way, leaders can stimulate their followers and their efforts are streamlined by realizing expectations vested on them, which, in turn, influence the performance of the business in a positive direction at levels of operational stages. With the help of intellectual stimulation, executives motivate employees to question communicated assumptions and improve problem-solving and discussion culture for attaining intellectual development and innovation, and that helps in shaping the better performance of the company (Deinert et al., 2015). In addition, the CEOs, through individualized consideration, can achieve better performance of the business when they consider the needs of individuals and support them for personal growth and development (Crawford et al., 1997). Individualized consideration positively influences the performance of employees that, in return, improves the overall performance of the business (Kim and Shin, 2019). One more theoretical approach contextualizes leadership impacts on business efficacy: “The higher echelons theory” (Hambrick and Mason, 1984). According to this strategy, the top executives in business extensively impact business outcomes. Another study concludes that the charismatic personality of a leader, or idealized influence, greatly differs in performance and outcomes (Waldman et al., 2004).

In contrast, it is of great consideration that some of the researchers failed to demonstrate an association between CEO transformational style and business outcomes specifically, where idealized influence prevailed (Samson and Ilesanmi, 2019). Similarly, some scholars raise queries regarding the use of subjective examination measures for leadership by distributing questionnaires to the followers (Ingram, 2016). They recommended using objective measures other than the perceptions of the followers regarding their leaders in the examination of leadership style for validating the theory implications (Antonakis and House, 2014). Accordingly, the hypothesis proposed is:

Hypothesis 1: Transformational leadership has a significant influence on firm performance.

#### Mediating Role of Corporate Sustainability

Doh and Quigley (2014) described leadership as an executive position in a firm with the ability to influence others. In contrast, Opoku et al. (2015) shared the opinion that leadership is not only required at all stages in an organization, but can also emerge at various organizational levels. Moreover, leadership is not concerned with any specific position in an organization, but it can be experienced by different officials at different stages of operations in an organizational context (Bass and Riggio, 2006). Furthermore, Ferdig (2007) explained that leaders are the persons who inspire others, share vision, develop harmony, guide followers, and transform changes in the values and activities of the subordinates to achieve organizational goals. In the context of corporate sustainability, communication of organizational vision by the leaders is welcomed by all stakeholders as reliable information (Linnenluecke and Griffiths, 2010). Accordingly, corporate sustainability can be defined as “the leadership and management notions that a corporation embraces, so that it can deliver social, environmental, and economic outputs at the same time” (Linnenluecke and Griffiths, 2010). So, members of a business, who take the role of leaders, can safeguard the interest of a business only if they effectively communicate the better future perspective of business to the firm, to themselves, and to the community (Wales et al., 2013). The relation of corporate sustainability to the performance of the business is gaining wider attention in corporate context because stakeholders are taking a keen interest in the responsibility culture of the organization (Eccles et al., 2014).

Moreover, this topic gained the extensive interest of researchers in evaluating the corporate sustainability impact on the performance of the organization (Burhan and Rahmanti, 2012). Researchers applied different methods to evaluate corporate sustainability including content analysis based on reports published about corporate sustainability, interviews, questionnaires, and various indexes (Aggarwal, 2013). Accordingly, López et al. (2007) studied the association between corporate sustainability reporting about the economic performance of the business and concluded a significant positive association among these. Schadewitz and Niskala (2010) conducted further research based on Finland organizations by applying the GRI reporting framework. This research concluded that quality disclosure of corporate sustainability has a significantly positive connection with the organization’s market value. In their study, Reddy and Gordon (2010) found a significant association of corporate sustainability reporting in describing abnormal profits by applying the “event study method” taking a sample of 68 corporations registered with the stock exchange of New Zealand and Australia. Similarly, Ameer and Othman (2012) concluded, while studying the qualitative features of CS reports about financial outcomes of business, that a correlation exists among variables. They incorporated data from the top 100 sustainable organizations around the globe for examination. Hence, the above-discussed literature indicates that corporate sustainability potentially mediates the association between transformational leadership and the performance of the business. Accordingly, the hypotheses proposed are:

Hypothesis 2: Transformational leadership has a significant influence on corporate sustainability.

Hypothesis 3: Corporate sustainability significantly mediates the association between transformational leadership and firm performance.

#### Knowledge-Sharing as Moderator

Knowledge management is defined by researchers as the acknowledgment and application of obtained information in a business to counter competitors (Alavi and Leidner, 2001). Dissemination and availability of knowledge is an integral part of knowledge management between organizations or within the business. In the literature, it is described as the communication of collected information and capabilities while resolving issues, developing innovative ideas, and applying policies and procedures (Chen et al., 2018). Some other scholars defined the concept as a process consisting of various stages, including commencement, application, approval (Yu and Yang, 2018), communication (Cavusgil et al., 2003), or sharing and integration (Wang, 2019). In the past, the concept of knowledge-sharing was surprisingly neglected by people (Cummings, 2004). However, at the start of the 20th century, the significance of knowledge-sharing was acknowledged by humans over time. After that, knowledge management and its processes remained a concept of core importance in the area of human resources (Blankenship and Ruona, 2009). Macneil (2001) particularly paid importance to tacit knowledge and considered it the most valuable kind of knowledge that includes expertise, abilities, and understanding of humans. Consequently, participants are motivated to exercise both kinds of knowledge, i.e., explicit and tacit, with the help of knowledge-sharing culture, while facing a problematic situation (Lin and Lee, 2004). Another study concludes that knowledge-sharing more effectively affects the ambidexterity of employees while working (Caniëls et al., 2017; Caniëls and Veld, 2019).

“Knowledge-sharing” is a complex idea. Knowledge is a priceless asset that could generate extra value for a business for its advancement (Jeon et al., 2011). Additionally, sharing of knowledge is concerned with the assimilation and integration of information (Nooteboom et al., 2007). Chiu et al. (2006) indicated that sharing information helps an organization when allocating resources. In numerous organizations, it is proven that knowledge is a substantial source that performs a decisive role in the long-term performance of an entity (Choi et al., 2008). Obtaining a sustainable advantage is based on the capability of a business to create and implement intellectual information. In addition, Jilani et al. (2020) concluded that attaining and practicing an effective knowledge management environment consistently results in the better performance of the organization. Likewise, Hoopes and Postrel (1999) argued that the development of a distinctive knowledge communication framework with an integrated approach potentially provides sources of competitive edge and, thus, enhances sustainable performance. Besides, human capital theory hypothesizes the impact of employees’ information, ability, skill, and other qualities about the sustainability of an organization (Schultz, 1961; Cummings, 2004). Based on the theory of human capital, this study hypothesizes that the behavior of sharing knowledge among participants can enhance and support the dynamic capabilities of an organization in developing sustainable operations (Lee and Ha-Brookshire, 2017). Therefore, this study theorizes that the knowledge-sharing process works as “enablers” that communicate information appropriately, which results in the sustainable performance of the business (Obeidat and Tarhini, 2016; Caniëls et al., 2017). Accordingly, it is hypothesized that:

Hypothesis 4: Knowledge-sharing significantly moderates the relationship of transformational leadership with corporate sustainability.

## Research Methodology

### Research Approach

Research methodology depends on the objective and problem of the study (Sabir et al., 2019), and a suitable methodology is essential for the accuracy of findings. By considering the problem and objective of the current study, a quantitative approach to research has been chosen and a cross-sectional method has been used for data. For data collection purposes, a questionnaire was used by the researchers. The use of a survey questionnaire is appropriate for the current research study because it makes it possible for researchers to collect data in a reasonable time and it is a cost-effective method of data collection (Sekaran and Bougie, 2003). Furthermore, this method ensured respondents’ secrecy and sensitive information can be easily collected. See Appendix.

### Questionnaire Development

The primary purpose of the research is to investigate the mediating role of corporate sustainability in the relationship of the impacts of transformational leadership on the performance of firms. This study also aimed to investigate the moderating role of knowledge-sharing on the relationship of transformational leadership with corporate sustainability. A questionnaire has 26 items that were all drawn from prior studies and rated on a 5-point Likert scale (ranging from 1, “strongly disagree,” to 5, “strongly agree”). A pilot study was conducted first to determine the questionnaire’s reliability and validity. The respondents reviewed the questionnaire and made some suggestions for improvements. The feedback and ideas of the pilot research respondents were heeded, and the resulting instrument was tweaked and refined before being delivered to the study’s target population for data collection. The scales items were adapted from existing studies. Items for firm’s performance were adapted from the study of Hancott (2005), the scale for Knowledge-sharing was adapted from the study of Jilani et al. (2020), and the scale of transformational leadership was adapted from Vera and Crossan (2004).

### Sampling and Data Collection

The study’s target group included project managers, project team leaders, and project staff. We collected information from the largest automobile companies, such as the Shanghai Automotive Business Corporation (Group), China FAW Group Corporation, Dongfeng Motor Co., Ltd., Beijing Automotive Group Co., Ltd., and China North Industries Group Corporation. These companies have the biggest market share in the Automobile Manufacturing industry in China. Following research ethics, all participants were assured that the information they provided would be kept private and solely used for this study. The data for this study were collected using a convenient sampling technique from the respondents. This study used Krejcie and Morgan’s (1970) table for determining the study’s sample size. Furthermore, this study also used G*Power version 3.1 software to confirm the sample size. This study obtained a sample size of 98 at a statistical power of 0.95. The current study chose a sample size of 300. Moreover, Table 1 represents the response rate of distributed questionnaires.

**TABLE 1:** Demographics of respondents (*N* = 198).

Respondents’ profile	Categories	Percentages
Gender	Male	70.5
	Female	29.5
Age	20–30	10.5
	31–40	24.5
	41–50	30.5
	Above 50	34.5
Education	Intermediate	15.0
	Graduate	37.0
	Master	46.0
	Higher	2.0
Position	Top level	26.0
	Middle level	34.0
	Line manager	18.0
	Entry level	22.0
Income level	21–40K (Yuan)	16.5
	41–60K (Yuan)	23.5
	61–80K (Yuan)	27.0
	Above 80K (Yuan)	33.0

### Demographic Profile of the Respondents

Table 1 show the demographic profile of the respondents. A total of 198 individual respondents have answered the questionnaire and their responses were analyzed. The study reveals that men were 70.5%, whereas women are 29.5% of the total sample size. It can also be concluded from the analysis that the number of men participating in activities, such as in transformational leadership and firm performance, is greater than the number of women. Moreover, older respondents have more confidence as they gained more experience and insights over time, so they are more aware of the role of transformational leadership and firm performance. Respondents 20–30 years of age are 10.5%, while respondents 31–40, 41–50, and above 50 years of age are 24.5, 30.5, and 35.5%, respectively. A higher education gives a more optimistic outlook and a profound effect on employee performance as most of the graduates and postgraduate respondents are well-aware of the importance of transformational leadership and firm performance; the percentage levels of graduate and postgraduate respondents are 37 and 46%, respectively. Furthermore, most of the respondents are top-level and middle-level employees in various sectors. Due to their high job profile in their respective group, 26 and 34% represent a higher level of commitment toward transformational leadership and firm performance. Finally, respondents with income levels of 61–80K (Yuan) and above 80K (Yuan) ratio of their investment are 27 and 33%, respectively.

## Analysis and Results

This study used Smart PLS 3 (SEM) for data analysis and adopted a two-step technique to analyze data and in reporting of analysis results (Henseler et al., 2009).

### Measurement Model Assessment

For the examination of the reliability and validity of data, measurement model was used by using PLS-SEM (Ringle et al., 2015). The reliability of constructs was examined by the values of the factor loading, Cronbach’s alpha, composite reliability, and average extracted variance (AVE). Discriminant validity was also evaluated by using a measurement model (Fornell and Lacker, 1981). Table 2 illustrates the results of the measurement model.

**TABLE 2:** Internal consistency, convergent validity, composite reliability, and AVE.

Construct	Indicators	Loadings	Cronbach’s alpha	Composite reliability	AVE
Firm performance	FP1	0.731	0.919	0.934	0.614
	FP2	0.688			
	FP3	0.627			
	FP4	0.664			
	FP5	0.845			
	FP6	0.880			
	FP7	0.867			
	FP8	0.849			
	FP9	0.854			
Corporate sustainability	CS1	0.809	0.895	0.923	0.706
	CS2	0.878			
	CS3	0.904			
	CS4	0.848			
	CS5	0.754			
Knowledge-sharing	KS1	0.816	0.844	0.889	0.618
	KS2	0.881			
	KS3	0.793			
	KS4	0.736			
	KS5	0.691			
Transformational leadership	TL1	0.737	0.888	0.913	0.601
	TL2	0.832			
	TL3	0.869			
	TL4	0.816			
	TL5	0.693			
	TL6	0.720			
	TL7	0.753			

*Source: Authors’ estimations based on data.*

SmartPLS is used to analyze data using two basic techniques: partial least squares and structural equation modeling (Shiau et al., 2019). SmartPLS is an obvious choice for diverse transformational leadership and firm performance when the objective of the study is to reveal such a relationship and make predictions in the model. Table 2 shows the results of two measurement model components: convergent validity and internal consistency reliability. All the components and indicators have to meet the model’s specific measuring requirements. The outer loading values of the model exceeded the specific criterion of 0.650, suggesting that indicator reliability has been achieved (Shiau et al., 2019). Furthermore, the recovered average variance value is bigger than the target value (0.50) suggesting that the model’s conversion validity has been realized (Shiau et al., 2019). Furthermore, the composite reliability values vary from 0.889 to 0.934, suggesting that the values are greater than 0.70, implying that internal consistency has been achieved (Shiau et al., 2019). The results of the two tests show that the model’s measurements, such as convergent validity, internal consistency, and reliability, are perfect.

The heterotrait-Monotrait Ratio is a substitute technique to find out the construct’s discriminant validity. Discriminant validity refers to the degree of correlation between measurement items from one construct and measurement items from other unrelated constructs that should not be connected. This test determines how much variance can be attributed to a group of constructs where two “conceptually dissimilar” constructions must be sufficiently different (Henseler et al., 2012). The recommended value of HTMT is below 0.85 to achieve discriminant validity (Kline, 2011). Table 3 shows that the level of discriminant validity in the study is achieved.

**TABLE 3:** Heterotrait-Monotrait Ratio (HTMT).

	CS	FP	KS	TL
CS				
FP	0.574			
KS	0.545	0.670		
TL	0.531	0.479	0.431	

*Source: Authors’ estimations based on data.*

**FIGURE 1:**
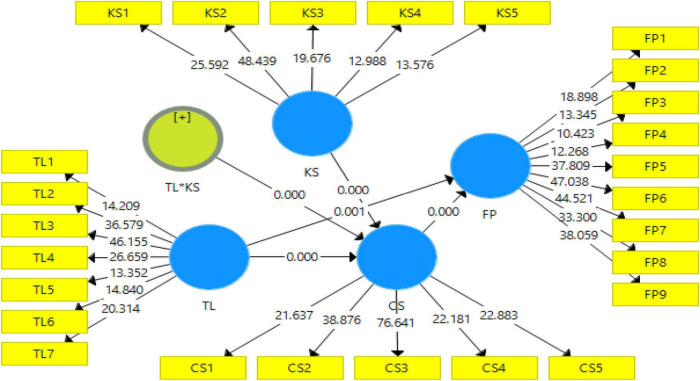
Structural model assessment.

Table 4 demonstrates that all values of VIF are lower than a threshold of 5 implying that there is not a problem with collinearity across the various constructs (Cheah et al., 2018). The modified *R*^2^, which presented the amount of variance explained by exogenous variables by endogenous variables, is used to determine the predictive power of the model. The modified *R*^2^ value of 0.580 implies that, overall, all behavioral practices contribute more than 58% of individual investment decisions. The findings of the size of the effect using the model’s *f*^2^ are shown in Table 4. Effect sizes with values ranging from 0.005 to 0.055 are included in this category. All the Q2 values in the model are < 0 indicating the reliability of the model. The goodness of fit values is 0.025 < 0.080. The value of the normal fit index is 0.732 and is close to 1, and the value of theta is close to 2 showing the reliability of the model fit with specified analysis. The structural model assessment is depicted in Figure 1.

**TABLE 4:** Structural model.

Pattern Rsqr	Adj	*f2*	Q^2^	VIF	SRMR	NFI	rms Theta
CS	–	0.055	0.040	2.320	–	–	–
FP	–	0.005	0.028	2.201	–	–	–
KS	–	0.048	0.043	2.534	–	–	–
TL	0.580	–	–	–	0.025	0.732	0.149

### Structural Model Assessment

The structural model assessment (Direct Effect) is shown in Table 5. For the estimation of the hypotheses of the study, SEM-PLS structural model analysis was conducted. The first hypothesis of the study states that transformational leadership has a significant positive relationship with the performance of the firm. The results of the structural model analysis show that transformational leadership behavior plays a significant role in the performance of a firm (β = 0.233, *t* = 3.504). Therefore, H1 is supported by the results of the analysis. Moreover, the second hypothesis of the study states that corporate sustainability has a significant positive relationship with the performance of the firm. The results of the study reveal that corporate sustainability has a significant positive relationship with the performance of a firm (β = 0.420, *t* = 6.609) and H2 is accepted on statistical grounds.

**TABLE 5:** Structural model assessment (direct effect results and decision).

Hypotheses	Relationship	Beta	STDEV	T statistics	*P*-values
H1	TL→FP	0.233	0.067	3.504	0.001
H2	CS→FP	0.420	0.064	6.609	0.000

*Source: Authors’ estimations based on data.*

The structural model assessment (Indirect Effect) is shown in Table 6. For the estimation of the mediation effect, the bootstrapping procedure is adopted by using PLS-SEM. The third hypothesis of the study is that corporate sustainability positively mediates the relationship between transformational leadership and the performance of the firm. The results of the analysis indicated that corporate sustainability positively mediates the relationship between transformational leadership and the performance of the firm (β = 0.147, *t* = 4.567) and supported H3.

**TABLE 6:** Structural model assessment [indirect (mediation)].

Hypotheses	Relationship	Beta	STDEV	T statistics	*P*-values
H3	TL→CS→FP	0.147	0.032	4.567	0.000

*Source: Authors’ estimations based on data.*

Table 7 specified the results of the moderation analysis, according to the fourth hypothesis of the study that knowledge-sharing positively moderates the relationship between transformational leadership and the performance of the firm. The results of the analysis revealed that knowledge-sharing plays a significant and positive moderating role in the association of transformational leadership behavior with corporate sustainability (β = 0.349, *t* = 5.617) and supported H4.

**TABLE 7:** Structural model assessment (moderation effects).

Hypotheses	Relationship	Beta	STDEV	T Statistics	*P*-values
H4	TL*KS→FP	0.349	0.062	5.617	0.000

Source: Authors’ estimations based on data. *Denote the moderating relationship of Knowledge sharing and Transformational leadership with firm performance.

### Goodness-of-Fit Index

The geometric mean of both the AVE and the endogenous variables’ average *R*^2^ is used as the global fit measure (Tenenhaus et al., 2005). The index shows whether the model is fit completely to explain the data. Its values range between 0 and 1. The values close to 1 show a strong model fit (Wetzels et al., 2009). Calculations are provided in Table 8. The table below shows the Goodness-of-fit (GOF) value of 0.450, which shows a strong model fit. Our model is fit to explain comprehensively the prediction of the data in the analysis.

**TABLE 8:** Goodness-of-fit index calculation.

Construct	AVE	R2
CS	0.45	0.16
FP	0.44	0.14
KS	0.27	0.26
TL	0.31	0.21
AVE × R2		
GOF = √(AVE × R2)	0.450	

## Discussion

The primary purpose of the research is to investigate the mediating role of corporate sustainability in the relationship between the impacts of transformational leadership on the performance of firms. This study also aimed to investigate the moderating role of knowledge-sharing on the relationship of transformational leadership with corporate sustainability. Transformational leaders influence the performance of their followers by strengthening social relationships (Wang et al., 2016). TL strengthens the emotional bond or identification between the supervisor and the follower, allowing the follower to perform above and beyond expectations. The successful adaptation of organizations to their new environment necessitates the involvement of more transformational leaders. Transformational leaders may successfully alter an organization’s culture and build a system-wide alignment of the organization’s strategies to meet the demands of the environment. Furthermore, economic, social, and environmental sustainability are the three pillars of corporate sustainability that work together to help businesses achieve more sustainable practices. Businesses must change their mindset from one of quick profits at the expense of the environment to one of mutual interdependence and eco-innovation. The adopting of sustainable practices benefits businesses in a variety of ways including greater brand image, lower costs, happier shareholders, more production, and a slew of other advantages. Individuals, businesses, and governments are all prioritizing sustainability as a critical component of their strategies. At a time when society is becoming more conscious of its impact on the environment, the corporate landscape is undergoing significant changes because of this collective push toward a more sustainable future. Finally, organizations may use knowledge-sharing to improve their skills and capabilities, raise their value, and maintain their competitive advantage. Knowledge is a company’s most important resource since it embodies intangible assets, routines, and creative processes that are difficult to duplicate (Renzl, 2008). Knowledge-sharing enhances the knowledge resource, and dynamic capability plays a significant part in achieving a competitive advantage (Choi et al., 2017). Researchers discovered that an organization’s inventive capability increases its energy level, which has a beneficial impact on performance (Proksch et al., 2017).

## Conclusion

The purpose of this study is to inspect the role of transformational leadership in firm performance with a mediating effect on corporate sustainability. This study also intended to explore the moderated mediation effect of corporate sustainability with the moderating role of knowledge-sharing. The findings of the analysis indicated that transformational leadership has a positive and significant association with firm performance. Sleiman (2016) and Jensen et al. (2020) argued that the behavior of transformational leadership has a significant influence on the performance of the firm. However, transformational leadership depends on the sector, and it confers sustainable competitive advantage in a competitive environment. Moreover, the results of the analysis revealed that corporate sustainability has a significant mediation role in the association of transformational leadership with firm performance. According to Eccles et al. (2014), those companies that are more sustainable outperform in terms of accounting performance and the stock market in the short, as well as in the long term. Findings also show that knowledge-sharing significantly moderates the relationship of transformational leadership with corporate sustainability.

### Theoretical Implications

The roles of transformational leadership have been studied in a broader context in prior studies; this research study will add to the current literature to inspect the role of transformational leadership in firm performance with the mediating effect of corporate sustainability with the moderating role of knowledge-sharing. Moreover, there has been minimal research on the role of transformational leadership in firm performance with mediating effect of corporate sustainability with moderating role of knowledge-sharing particularly in the automobile sector in a developed country such as China. This study has demonstrated the importance of various leadership styles and their significant impacts on firm performance. Corporate sustainability has a significant mediating role in the association of transformational leadership and firm performance. Findings indicated that knowledge-sharing has a moderating role in the association of transformational leadership and firm performance. The findings of this study contribute to the body of knowledge and show that leadership style has a significant effect on firm performance, and knowledge-sharing culture in firms is essential for better performance of the firm. Furthermore, firms may improve their performance by improving their sustainability and by creating a knowledge-sharing culture.

### Practical Implications

From a practical perspective, the finding of the contemporary study has several implications for top management in the automobile sector. Leadership style plays an important role in the performance of a firm and knowledge-sharing is the vital component that enhances the positive effect of transformational leader behavior on firm performance. Top management of firms needs to develop transformational leader behavior and create a knowledge-sharing environment in the firm for better performance in a competitive environment. Furthermore, firms may improve their performance by improving corporate sustainability.

### Limitations and Future Research Directions

There are various limitations in this study that need to be addressed in future research. Firstly, the study’s sample was limited to individuals working in China’s small- and medium-sized automobile sector, which may make the findings hard to extend to new businesses in a new production set up. Secondly, this study only considered a small sample of individuals from a specific area in China and ignored the rest of the country. The third limitation of the study is about the many forms of leadership selected to represent the idea of management leadership as the literature lists various other types of leadership besides leadership that is both revolutionary and transformational. Furthermore, the study focused on the impact of styles of leadership, such as revolutionary and transformational, on specific corporate sustainability practices, ignoring other processes that may be important to the organization. Fourth, corporate sustainability and firm performance may not be solely determined by leadership. Other factors may be important in understanding this relationship and should be considered. Fifth, the primary data collection approach was a quantitative methodology, which may be viewed as a study limitation. Questionnaires and other self-reporting data collection procedures may cause prejudice in response; consequently, to achieve the study goals’, more qualitative techniques should be used to collect more accurate information and results. Finally, this study was conducted in the context of Chinese culture setup so the findings may be limited to the belief, ethics, and values of the Chinese working environment.

Future research should replicate our findings across a sample of diverse organizations, so that new businesses could be accurately signified (Wales et al., 2013). Furthermore, the forthcoming study should attempt to collect samples from other regions around the country to improve the generalizations of the study. Moreover, if the researcher measured the different constructs of the study while using different dimensions it will be interesting to see whether the results were matching or were different from the prior studies. Future studies should be aware of this link and may attempt to provide further discernment by considering new characteristics in addition to leadership styles that may have an impact on the success of leaders in organizations and other structures in the future. Similarly, future studies should be directed to use mediating and moderating relationships to the literature and provide further explanation. To get data that accurately represents the study’s variables, researchers should use both quantitative and qualitative data collection methodologies. Future researchers should use structural equation modeling as an analytical technique because it is thought to be the most effective at streamlining the embellishment of the basic model of the study.

## Data Availability Statement

The original contributions presented in the study are included in the article/supplementary material, further inquiries can be directed to the corresponding author.

## Author Contributions

MS presented the main idea and contributed to writing the draft. TI contributed to the technique and methodology. NJ collected the data. MZ performed the analysis. All authors contributed to the article and approved the submitted version.

## Conflict of Interest

The authors declare that the research was conducted in the absence of any commercial or financial relationships that could be construed as a potential conflict of interest.

## Publisher’s Note

All claims expressed in this article are solely those of the authors and do not necessarily represent those of their affiliated organizations, or those of the publisher, the editors and the reviewers. Any product that may be evaluated in this article, or claim that may be made by its manufacturer, is not guaranteed or endorsed by the publisher.

## Appendix A

### Questionnaire of the Study

#### Firm Performance

1. Firm Performance is the extent to which individuals or organization remains successful while achieving a mission.
2. Firm Performance is the depiction of several fulfilled tasks and set targets or the aim of business at the end.
3. Firm performance is the ability of firms in using human and material resources to achieve their targets.
4. The success of the firm’s performance depends upon the capabilities of the organization personnel.
5. CEOs potentially form the performance of their organization by communicating its core values and beliefs.
6. Transformational leadership and job satisfaction have a significant relationship with firm performance.
7. Organizations structure of command is the key driver for better performance in the market.
8. Organizations in challenging environments show strong commitment regarding the achievement of their targets.
9. Organizational pay consideration is crucial for individuals for achieving firm-specific performance.

#### Transformation Leadership

Following rules should neither be corrupted nor self-serving.

1. Go beyond self-interest for the bettering and effectiveness of firm performance.
2. Never take the achievements of other people as his/her own.
3. Explain the long-term meaning of the performance to personnel.
4. Consider the real conditions of personnel when in contact with them.
5. Show high commitment to his/her work and keeps high levels of passion while facilitating personnel.
6. Good at and never hesitate to take action when dealing with difficult circumstances.

#### Corporate Sustainability

1. We know enough about corporate sustainability.
2. Organizations, where operations are based on sustainable growth, social responsibility, and environmental protections, are sustainable.
3. Sustainable organizations would be considered superior firm performance as the essential component of organizational strategy.
4. Sustainable organizations exploit environmental challenges and legislations to their advantage by developing new environmentally sustainable performances in the organization.
5. Corporate sustainability must be taken as an important route for the long-term development of firm performance.

#### Knowledge-Sharing

1. Knowledge management is the acknowledgment and application of obtained information in a business to counter the competitors.
2. Dissemination and availability of knowledge is an integral part of knowledge management between organizations or inside the business.
3. Knowledge-sharing organizations better achieve their targets as compared to competitors in the market.
4. Organizational personnel are motivated to exercise both kinds of knowledge, i.e., explicit and tacit with the help of knowledge-sharing culture, while facing a problem situation.
5. Getting a sustainable advantage based on the capability of a business of creating and implementing intellectual information.
